# Supplementary material for: TGF-β Type I Receptor Signaling in Melanoma Liver Metastases Increases Metastatic Outgrowth
Source: Int J Mol Sci. 2023 May 12;24(10):8676. doi: 10.3390/ijms24108676 (PMC10218053; doi:10.3390/ijms24108676)
Supplement: Supplementary file 1 [file ijms-24-08676-s001.zip › Supplementary Tables S1 and S2.pdf]

Supplementary Table S1, cloning primers

Primer	Use	Sequence (5' – 3')
hALK5 FW bmtl	PCR	TAAGTAGCTAGCTGCAGATGGAGGCGGGG
hALK5 FLAG-age1 REV	PCR	TGTTAACGACCGGTGAGCGCTG
hALK5 FW	PCR	CGGATCCTTAGTGGTGG
hALK5 REV	PCR	ATTCAGTCGACTGATGGAGGCGGCGGTCGCT
kiALK5 K232R	Mutagenesis	GAAGAAGTTGCTGTTAGGATATTCTCCTCTAGAG
hALK5 FW seq	Sequencing	CAACCGCACTGTCATTCACCATC
hALK5 REV seq	Sequencing	TTCTTCTCCCCGCCACTTTCCTCT

Supplementary Table S2, rt-qPCR primers

Gene	Forward (5' – 3')	Reverse (5' -3')
hALK5	CAACCGCACTGTCATTCACCATC	TTCTTCTCCCCGCCACTTTCCTCT
mAlk5	ATTCCTCGAGACAGGCCATT	CAGCTGACTGCTTTTCTGTAGT
Serpine-1	GCCAACAAGAGCCAATCACA	AGGCAAGCAAGGGCTGAAG
Ctgf	GGCCTCTTCTGCGATTTCG	CCATCTTTGGCAGTGCACACT
Smad7	TGGATGGCGTGTGGGTTTA	TGGCGGACTTGATGAAGATG
Gapdh	CCAAGTCGGATGTGGAAATGG	TGTCGCAAGTGGACAGTCTC
Hprt	TGGATACAGGCCAGACTTTGTT	CAGATTCAACTTGCGCTCATC
