# Supplementary figures and images for: TGF-β Type I Receptor Signaling in Melanoma Liver Metastases Increases Metastatic Outgrowth
Source: Int J Mol Sci. 2023 May 12;24(10):8676. doi: 10.3390/ijms24108676 (PMC10218053; doi:10.3390/ijms24108676)

A

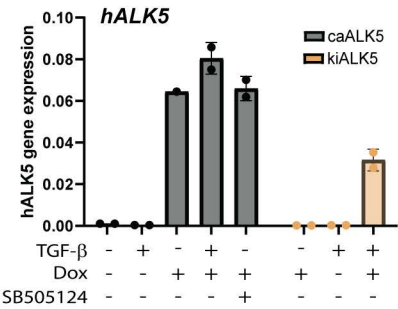

B

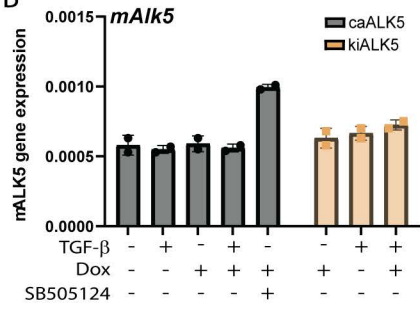

C

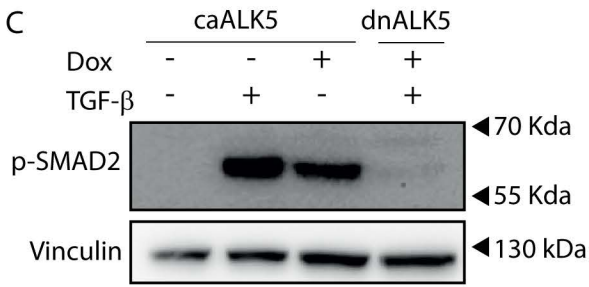

D

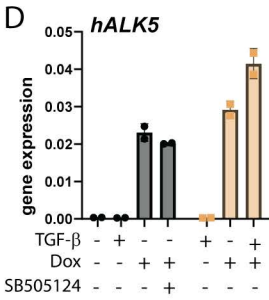

E

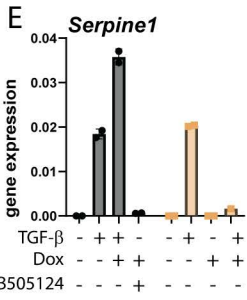

F

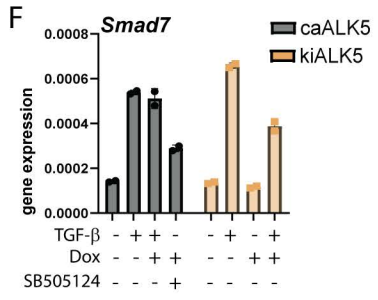

Supplement: Supplementary file 1 [file ijms-24-08676-s001.zip › Sup Figure S1.pdf]

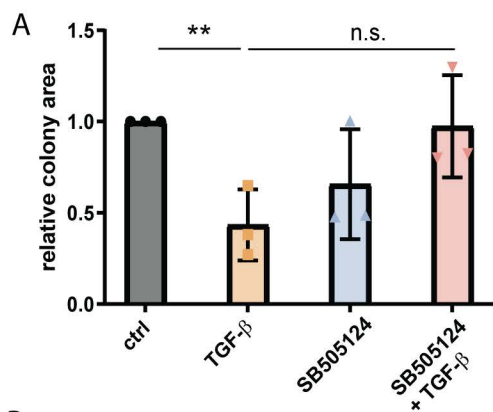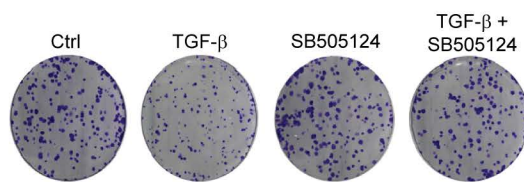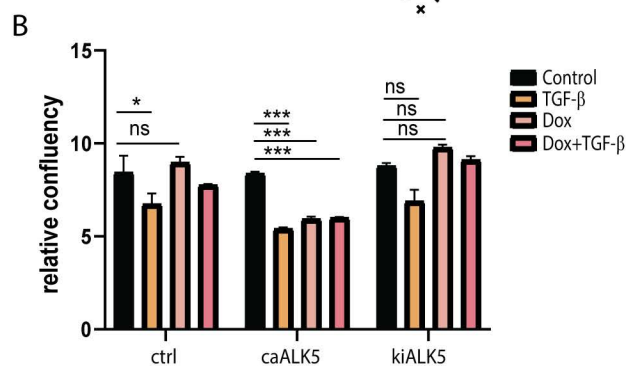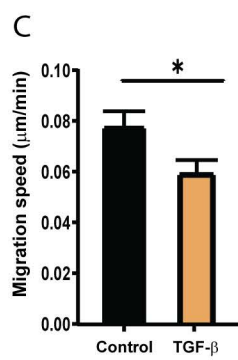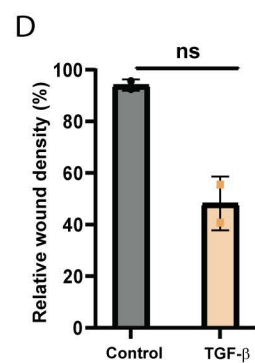

Supplement: Supplementary file 1 [file ijms-24-08676-s001.zip › Sup Figure S2.pdf]

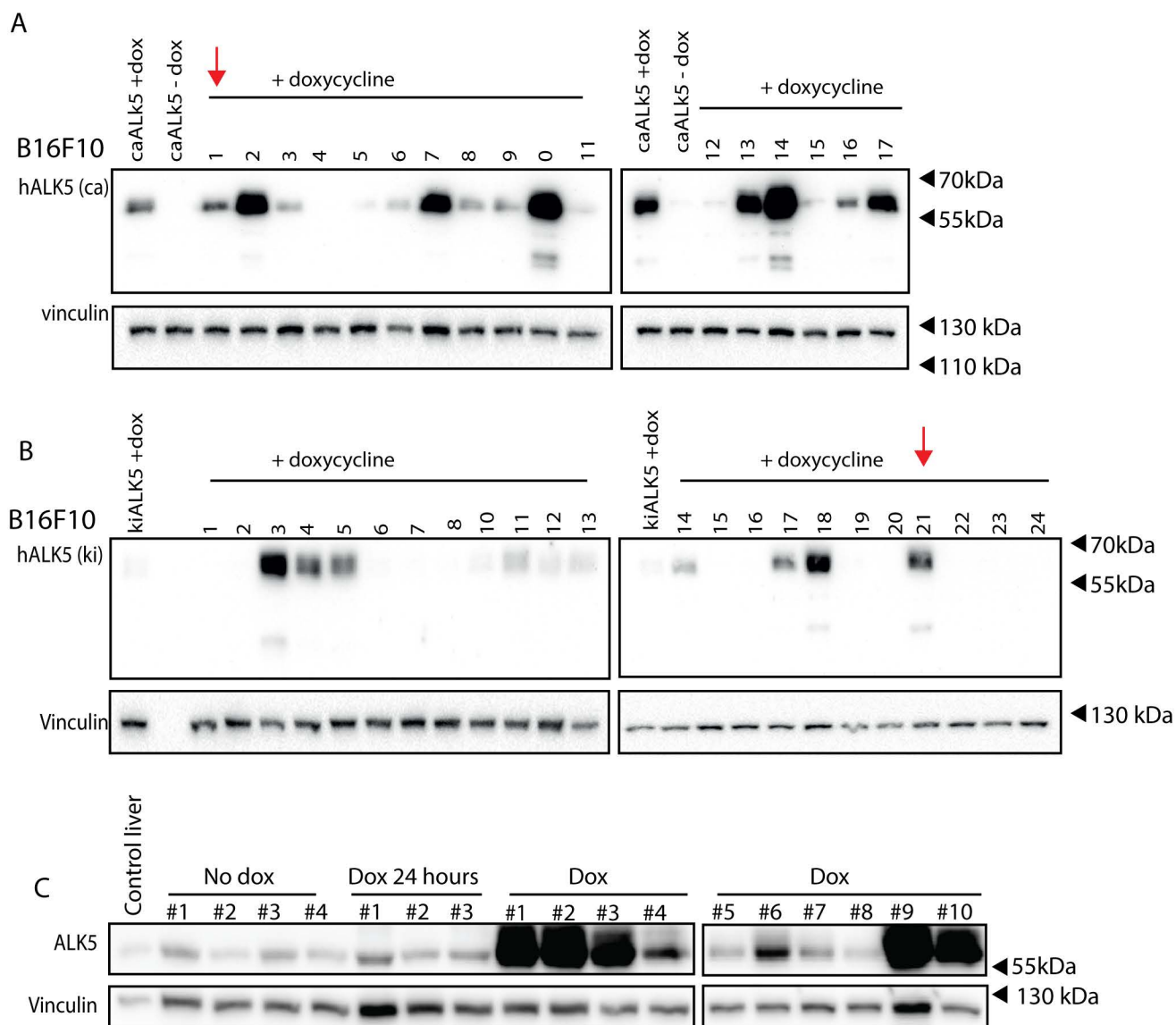

Supplement: Supplementary file 1 [file ijms-24-08676-s001.zip › Sup Figure S3.pdf]

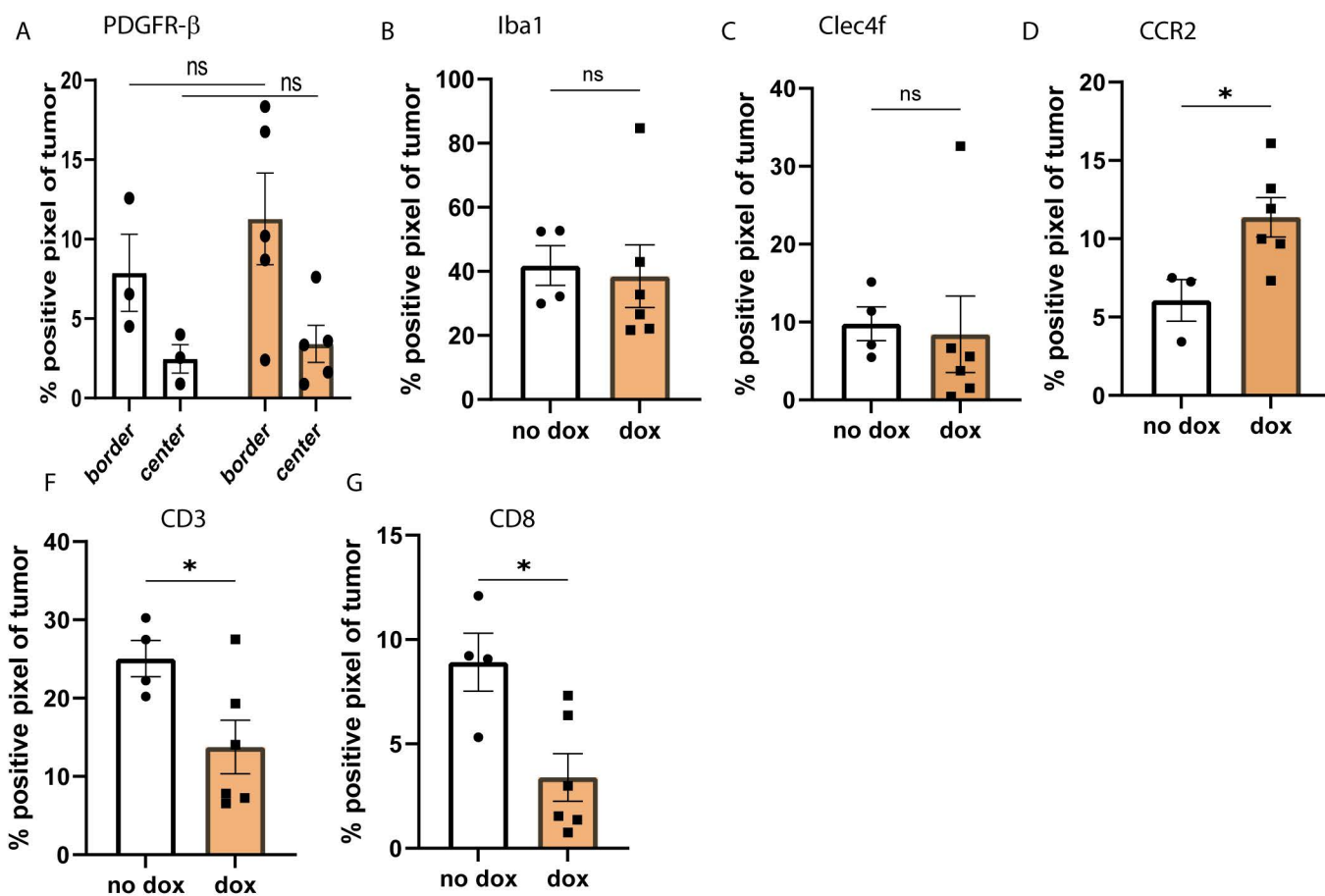

Supplement: Supplementary file 1 [file ijms-24-08676-s001.zip › Sup Figure S4.pdf]

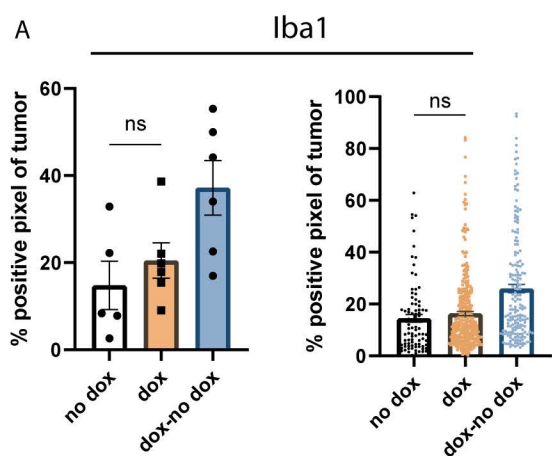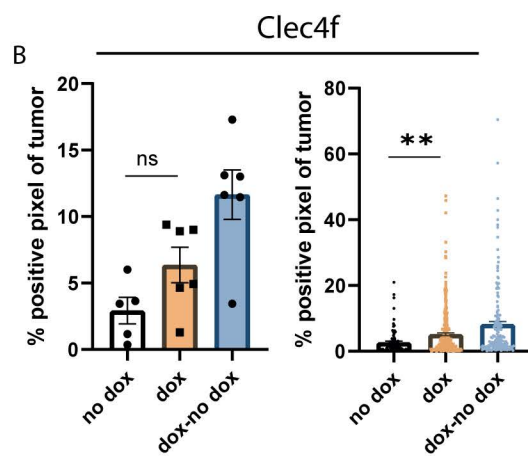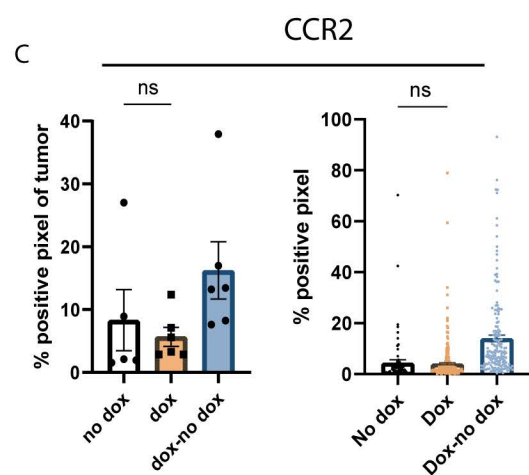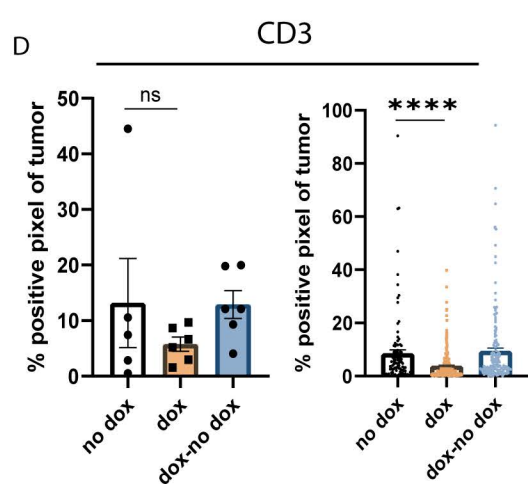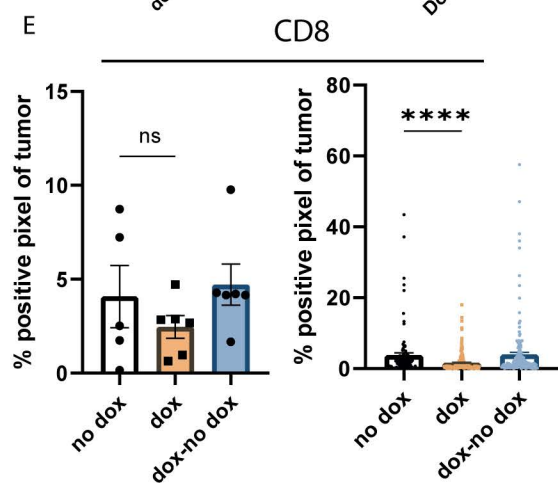

Supplement: Supplementary file 1 [file ijms-24-08676-s001.zip › Sup Figure S5.pdf]
